# Endothelial glycocalyx-associated molecules as potential serological markers for sepsis-associated encephalopathy: A systematic review and meta-analysis

**DOI:** 10.1371/journal.pone.0281941

**Published:** 2023-02-21

**Authors:** Sheon Baby, Tea Reljic, Nuria Villalba, Ambuj Kumar, Sarah Y. Yuan

**Affiliations:** 1 Morsani College of Medicine, University of South Florida, Tampa, FL, United States of America; 2 Department of Evidence Based Medicine, University of South Florida Morsani College of Medicine, Tampa, FL, United States of America; 3 Department of Molecular Pharmacology & Physiology, University of South Florida Morsani College of Medicine, Tampa, FL, United States of America; 4 Department of Surgery, University of South Florida Morsani College of Medicine, Tampa, FL, United States of America; Medical College of Georgia, Augusta, UNITED STATES

## Abstract

**Background:**

Sepsis-associated encephalopathy (SAE) is characterized by a diffuse cerebral dysfunction that accompanies sepsis in the absence of direct central nervous system infection. The endothelial glycocalyx is a dynamic mesh containing heparan sulfate linked to proteoglycans and glycoproteins, including selectins and vascular/intercellular adhesion molecules (V/I-CAMs), which protects the endothelium while mediating mechano-signal transduction between the blood and vascular wall. During severe inflammatory states, components of the glycocalyx are shed into the circulation and can be detected in soluble forms. Currently, SAE remains a diagnosis of exclusion and limited information is available on the utility of glycocalyx-associated molecules as biomarkers for SAE. We set out to synthesize all available evidence on the association between circulating molecules released from the endothelial glycocalyx surface during sepsis and sepsis-associated encephalopathy.

**Methods:**

MEDLINE (PubMed) and EMBASE were searched since inception until May 2, 2022 to identify eligible studies. Any comparative observational study: i) evaluating the association between sepsis and cognitive decline and ii) providing information on level of circulating glycocalyx-associated molecules was eligible for inclusion.

**Results:**

Four case-control studies with 160 patients met the inclusion criteria. Meta-analysis of biomarkers ICAM-1 (SMD 0.41; 95% CI 0.05–0.76; p = 0.03; I^2^ = 50%) and VCAM-1 (SMD 0.55; 95% CI 0.12–0.98; p = 0.01; I^2^ = 82%) revealed higher pooled mean concentration in patients with SAE compared to the patients with sepsis alone. Single studies reported elevated levels of P-selectin (MD 0.80; 95% CI -17.77–19.37), E-selectin (MD 96.40; 95% Cl 37.90–154.90), heparan sulfate NS2S (MD 19.41; 95% CI 13.37–25.46), and heparan sulfate NS+NS2S+NS6S (MD 67.00; 95% CI 31.00–103.00) in patients with SAE compared to the patients with sepsis alone.

**Conclusion:**

Plasma glycocalyx-associated molecules are elevated in SAE and may be useful for early identification of cognitive decline in sepsis patients.

## 1. Background

Sepsis is defined as a life-threatening organ dysfunction resulting from a dysregulated host response to infection [1–3]. Sepsis is the leading cause of non-cardiac deaths in intensive care units [4]. It is a major public health concern accounting for $32, 421 in hospitalization per patient [5] and an estimated $20 billion in total annual US hospital costs [6, 7].

Sepsis-associated encephalopathy (SAE) is characterized by a diffuse cerebral dysfunction that accompanies sepsis in the absence of direct central nervous system (CNS) infection [8–11]. Clinical presentation of SAE ranges from cognitive decline and mild delirium to deep coma [8, 12]. Differential diagnosis of SAE is based on neurological exam of mental status, neck stiffness, motor responses, muscular strength, plantar and deep tendon reflexes, and cranial nerves as well as imaging, electroencephalogram (EEG), and cerebrospinal fluid (CSF) analysis [13, 14]. However, bedside detection of cognitive performance is often limited by patient sedation [15].

SAE affects up to 76% of sepsis survivors [16]. Patients with sepsis with SAE exhibit significantly higher APACHE II and SOFA scores as well as 28- and 180-day mortality compared to patients with sepsis without SAE [17]. Thus, SAE is independently associated with increased mortality and poor outcomes such as long-lasting neurocognitive deficits [18, 19]. Despite increase in recognition, however, SAE has been understudied and its pathophysiology remains incompletely understood. Suggested mechanisms involve blood-brain barrier (BBB) breakdown, impairment of endothelial function, compromised cerebral autoregulation, and altered neurotransmission that ultimately results in neuronal dysfunction and cell death [20–25].

The vascular endothelium covering the luminal surface of blood vessels serves as a semi-permeable barrier that restricts the passage of circulating substances and cells from the blood to the tissues and is key in the regulation of vascular tone [26–28]. Protecting the apical surface of endothelial cells is the glycocalyx, a dynamic mesh comprised of membrane-attached proteoglycans and glycoproteins [29–31]. Proteoglycans, such as syndecans and glypicans, have a protein core and are covalently attached to one or more negatively charged glycosaminoglycans (GAGs) such as heparan, chondroitin, dermatan, and keratan sulfates [31, 32]. GAGs are long linear polymers composed of amino sugars and other sugars such as uronic acid and galactose [33]. Glycoproteins are short carbohydrate chains capped with sialic acid that mainly function as endothelial adhesion molecules such as selectins (E- and P-selectin), integrins, and immunoglobulins (ICAM and VCAM) [34].

Under inflammatory conditions, the endothelial cells become activated in response to proinflammatory stimuli (cytokines such as TNF-alpha) and increase the surface expression of E- and P- selectins, which facilitate leukocyte-endothelial interactions promoting rolling and initial attachment to the wall of postcapillary venules [35–37]. Subsequently, ICAM-1 and VCAM-1 mediate the firm adhesion of leukocytes to the endothelium followed by transmigration [36, 38]. ICAM-1 and VCAM-1 also play an active role in inflammation by increasing vascular permeability to permit solute and fluid exchange across inflamed microvessels [39]. These adhesion glycoproteins are upregulated in endothelial cells in response to cytokines or endotoxins [38, 40]. This sequence of events leads to leukocyte diapedesis into the inflamed tissues where they undergo chemotaxis towards invading pathogens or injured cells [36, 41].

During severe inflammatory states, proteoglycans, glycoproteins and GAGs, are shed into the circulation, some of which can be detected in soluble forms or as low molecular weight fragments [42]. The degree of glycocalyx damage and concentration of circulating glycocalyx-degradation products in the blood have been recognized as a marker of endothelial dysfunction following sepsis [29, 43–45]. Moreover, the injury to the glycocalyx and the release of inflammatory mediators contribute to a number of clinical manifestations of sepsis, including acute kidney injury, respiratory failure, coagulopathy and septic cardiomyopathy [46].

It has been shown that certain glycoprotein or proteoglycan fragments, including the extracellular domains of CD44 and syndecans, are highly capable of targeting endothelial junctions and promoting microvascular leakage [47]. Importantly, circulating glycocalyx products as a consequence of lung injury have been found to penetrate the hippocampal BBB during sepsis leading to cognitive dysfunction [48]. Additionally, increased concentration of sICAM-1 has been associated with vascular cognitive impairment in older adults [49]. The glycocalyx of the specialized brain endothelial cells acts as an additional layer together with other components of the BBB, synergistically regulating the trafficking of potentially harmful substances triggered by the inflammatory response from entering the brain [50].

Currently, there is no hallmark biomarker for SAE, and SAE remains a diagnosis of exclusion [51]. Although previous studies and ongoing clinical trials on SAE examine biomarkers associated with neuronal cell death such as neuron specific enolase (NSE), central nervous system specific protein (S100β), glial fibrillary acidic protein (GFAP), and Tau protein [52–55], there is a lack of information on whether endothelium damage and specifically glycocalyx-associated degradation products are useful for early diagnosis of SAE.

The aim of this systematic review is to synthesize all available evidence on the association between glycocalyx-associated molecules and SAE in patients with sepsis.

## 2. Materials and methods

This systematic review was conducted following a prespecified protocol and is reported according to the Preferred Reporting Items for Systematic Reviews and Meta-Analyses (PRISMA) guideline (S1 Table).

### 2.1 Patient and public involvement

There is no patient or public involvement in this study.

### 2.2 Criteria for study selection

Any observational comparative study which: i) evaluated the association between sepsis and cognitive decline and ii) provided information on level of glycocalyx-associated products were eligible for inclusion.

### 2.3 Search and study selection

We searched MEDLINE (PubMed) and EMBASE databases on 05/02/2022 using the search strategy (S1 File). We did not limit the search by date or language. The title and abstract of each reference were reviewed for initial screening. References were excluded if they did not include patients with SAE, did not evaluate glycocalyx-associated biomarkers, were not performed in humans, did not describe a clinical study (review, editorial, etc.), or were not in English. Reference lists were searched. Two authors (SB and TR) independently conducted the search and selected studies. Any disagreement was resolved by a third author (NV).

### 2.4 Data collection and management

Two authors (SB and TR) performed the data extraction using a standardized data extraction form. Any disagreement between the two authors was resolved by a third author (NV). We collected data on: study characteristics (study type, arms, location, funding, objectives, and patient enrollment), patient characteristics (age, gender, setting, etiology of sepsis, disease severity, admission characteristics, underlying disease, neurological characteristics), and levels of glycocalyx-associated products. When data were not extractable from the included reference, an attempt was made to contact the corresponding author.

### 2.5 Risk of bias assessment

Risk of bias was assessed using the Newcastle Ottawa Tool for Case-Control Studies. The assessment included the following three domains: selection, comparability, and exposure. For the selection domain, we examined if the case definition was adequate, representativeness of the cases, selection of controls, and definition of controls. For the comparability domain, we examined comparison of cases and controls. For the exposure domain, we examined the ascertainment of exposure, the method of ascertainment for cases and controls, and the nonresponse rate. For each criterion, the risk of bias was judged as low, high, or unclear.

### 2.6 Data analysis

When data were available from studies with similar populations and methods of measurement for exposures and outcomes, they were included in a meta-analysis. For continuous outcomes, mean and standard deviation were used to compute the mean difference for each study. We pooled the mean differences from individual studies using the random-effects model and reported the pooled mean difference, 95% CIs, and p-values. In the case where there was heterogeneity in the method of measurement but the population and outcome remained the same, we used the standardized mean difference to compute the pooled effect. Heterogeneity of pooled studies was evaluated using the I^2^ statistic. An I^2^<30% was considered low heterogeneity, I^2^<60% moderate heterogeneity, and I^2^≥60% high heterogeneity. To explore possible sources of heterogeneity of pooled studies and to test the robustness of the results, we performed a subgroup analysis according to definition of carcinoid crisis used by the authors.

Additionally, we performed a sensitivity analysis according to the timing of prophylaxis treatment.

All meta-analyses were conducted using STATA 16 [cite: StataCorp. 2019. Stata Statistical Software: Release 16. College Station, TX: StataCorp LLC].

## 3. Results

### 3.1 Results of search and study selection

**Fig 1:**
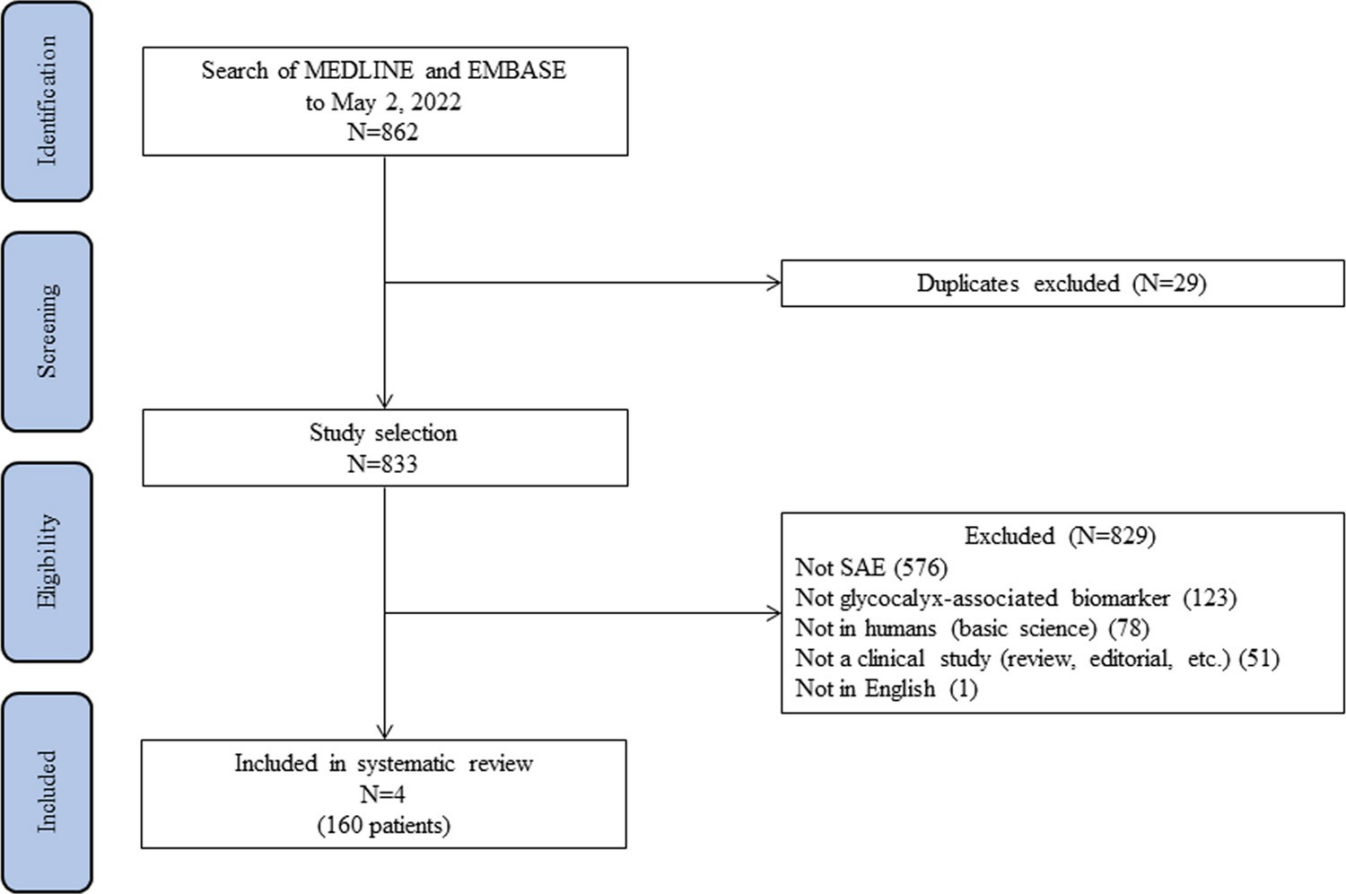
The PRISMA flow diagram. Our initial search yielded 862 citations, of which 29 duplicated were excluded. The abstracts of the remaining 833 citations were carefully checked and 829 were excluded. Ultimately, 4 studies were included in this analysis.

Our initial search yielded 862 citations, of which 29 duplicates were excluded (Fig 1). After a further review of the abstracts of the remaining 833 citations, 829 were excluded for various reasons: 576 citations were not about SAE, 123 citations were not about glycocalyx-associated biomarker, 78 citations were not in humans, 51 citations were not clinical studies, and 1 citation was not in English. Interestingly, 2 citations examined chondroitin sulfate. However, only the abstracts were available, and the results were pending publication. Ultimately, 4 studies met the inclusion criteria and were included in this analysis.

### 3.2 Characteristics of included studies

All included studies utilized a case-control study design and were published between 2009 and 2019. A total of 160 patients were included in these studies, of whom 105 and 55 patients had sepsis and SAE, respectively. SAE was diagnosed with Pediatric Glasgow Coma Scale (PGCS), Montreal Cognitive Assessment (MoCA), 2 or more presenting symptoms, or sepsis patients who later developed delirium. Three studies had adult patients while one study had pediatric patients (mean 4.31 years). In total, there were 97 males and 63 females. Disease severity was classified as sepsis or severe sepsis, with APACHE score, SOFA score, and/or CURB-65 score. The four studies were conducted in Egypt, Taiwan, Brazil, and United States with higher mortality in SAE group compared to sepsis group. See Table 1 for characteristics of included studies.

**Table 1:** Characteristics of included studies.

Study	N	Method of diagnosis for SAE	Age	Sex	Disease severity	Setting	Mortality
**Hamed et al. 2009**	Sepsis only (N = 24)	Pediatric Glasgow Coma Scale < 12	4.31 years	Male (N = 24)	Sepsis or severe sepsis	Pediatric Department, Egypt	N = 2, 5%
	SAE (N = 16)			Female (N = 16)			
**Hippensteel et al. 2019**	Sepsis only (N = 14)	Montreal Cognitive Assessment < 21	58 years	Male (N = 12)	APACHE III score (100)	ICU, University of Pennsylvania	---
	SAE (N = 6)			Female (N = 8)			
**Su et al. 2014**	Sepsis only (N = 47)	2 or more symptoms: somnolence, stupor, coma, confusion, disorientation, agitation, irritability, and decreased level of GCS.	Sepsis (62.6 years)	Male (N = 48)	• Severe sepsis and septic shock	Emergency Room, Taiwan	Sepsis only (N = 5, 11%)
	SAE (N = 23)		SAE (68.0 years)	Female (N = 22)	• APACHE II score: Sepsis only (17.5) and SAE (21.3)		SAE (N = 9, 40%)
					• SOFA score: Sepsis only (5.4) and SAE (8.2)		
**Tomasi et al. 2017**	Sepsis only (N = 20)	Sepsis patients who develop delirium within 3 days of hospital admission	Sepsis only (N = 20)	Male (N = 13)	CURB-65 score (2)	Respiratory Care Unit, Brazil	Sepsis only (N = 0, 0%)
	SAE (N = 10)		SAE (N = 10)	Female (N = 17)			SAE (N = 1, 10%)

All included studies utilized a case-control study design. A total of 160 patients were included in these studies, of whom 105 and 55 patients had sepsis and SAE, respectively. Three studies had adult patients while one study had pediatric patients (mean 4.31 years). There was higher mortality in SAE group compared to sepsis group.

### 3.3 Risk of bias in included studies

The overall risk of bias in included studies was judged as ‘low risk’ for most elements of the risk of bias assessment (Table 2). Two studies were judged to be at a high risk for bias regarding comparability of cases and controls since the studies do not distinguish the patient characteristics between each group [48, 56].

**Table 2:** Risk of bias assessment.

	Hamed et al. 2009	Hippensteel et al. 2019	Su et al. 2014	Tomasi et al. 2017
Is the case definition adequate?	Low risk	Low risk	Low risk	Low risk
Representative of the cases	Low risk	Low risk	Low risk	Low risk
Selection of controls	Low risk	Low risk	Low risk	Low risk
Definition of controls	Low risk	Low risk	Low risk	Low risk
Comparability of cases and controls on the basis of the design or analysis	High risk	High risk	Low risk	Low risk
Ascertainment of exposure	Low risk	Low risk	Low risk	Low risk
Same method of ascertainment for cases and controls	Low risk	Low risk	Low risk	Low risk
Nonresponse rate	Low risk	Low risk	Low risk	Low risk

The overall risk of bias was judged as ‘low risk’ for most elements of the assessment. Two studies were judged to be at a high risk for bias regarding comparability of cases and controls since the studies do not distinguish the patient characteristics between each group.

### 3.4 Outcomes

**Fig 2:**
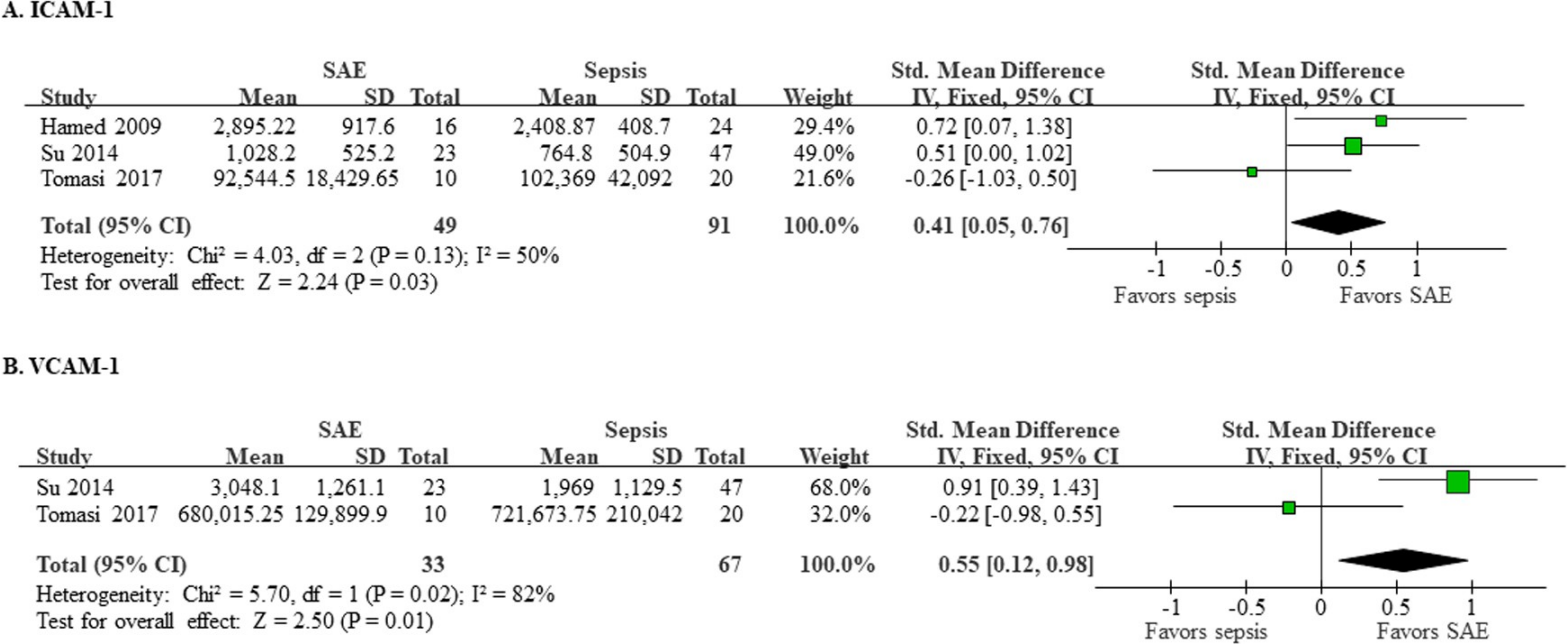
Levels of ICAM-1 and VCAM-1. Three studies (140 patients) reported data for ICAM-1 (A), and two studies (100 patients) reported data for VCAM-1 (B). The pooled mean concentration of ICAM-1 was higher in patients with SAE compared to the patients with sepsis alone (SMD 0.41; 95% CI 0.05–0.76; p = 0.03), and there was moderate heterogeneity (I^2^ = 50%) between studies. The pooled mean concentration of VCAM-1 was higher in patients with SAE compared to the patients with sepsis alone (SMD 0.55; 95% CI 0.12–0.98; p = 0.01), and the heterogeneity between studies was high (I^2^ = 82%).

Three studies (140 patients) reported data for ICAM-1 (Fig 2A). The pooled mean concentration of ICAM-1 was significantly higher in patients with SAE compared to the patients with sepsis alone (SMD 0.41; 95% CI 0.05–0.76; p = 0.03). The heterogeneity between studies was moderate (I^2^ = 50%). Regarding VCAM-1, two studies (100 patients) reported data (Fig 2B). The pooled mean concentration of VCAM-1 was significantly higher in patients with SAE compared to the patients with sepsis alone (SMD 0.55; 95% CI 0.12–0.98; p = 0.01). The heterogeneity between studies was high (I^2^ = 82%).

**Fig 3:**
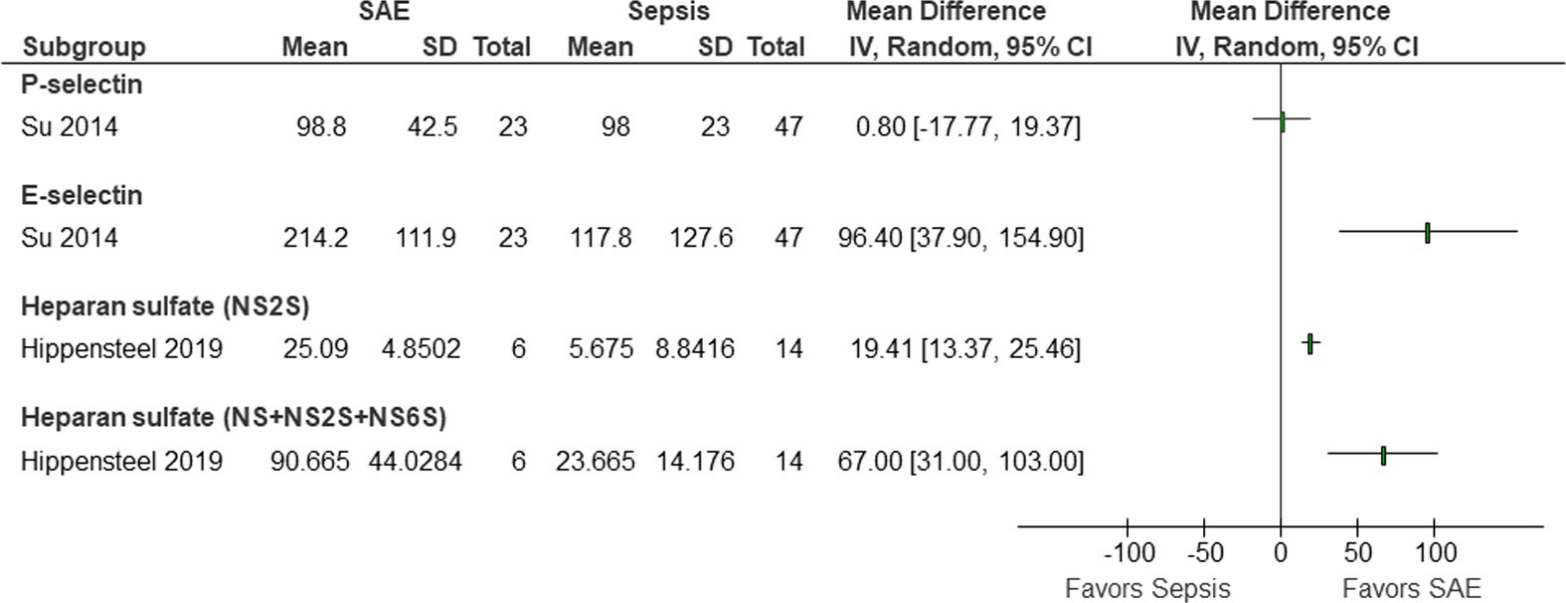
Levels of other biomarkers reported only by single studies. Single studies reported higher mean concentrations of P-selectin, E-selectin, and heparan sulfates in patients with SAE compared to patients with sepsis alone.

Other glycocalyx-associated biomarkers were reported by single studies (Fig 3), and therefore meta-analysis was not possible. Nevertheless, these studies reported mean concentrations higher in patients with SAE compared to the patients with sepsis alone. The mean difference of P-selectin, E-selectin, heparan sulfate (NS2S), and heparan sulfate (NS+NS2S+NS6S) were 0.80 (95% CI -17.77–19.37), 96.40 (95% Cl 37.90–154.90), 19.41 (95% CI 13.37–25.46), and 67.00 (95% CI 31.00–103.00), respectively.

## 4. Discussion

The findings from this systematic review and meta-analysis with 4 studies and enrolling a combined total of 160 patients show significantly increased levels of circulating heparan sulfate fragments and glycoproteins (ICAM-1, VCAM-1, P-selectin, and E-selectin) in sepsis patients with SAE compared to sepsis patients without SAE. A recent study by Hippensteel et al. [48] included 20 patients and proposed that circulating heparan sulfate fragments, as a consequence of glycocalyx degradation, accumulate in the hippocampus and inhibit brain-derived neurotrophic factor (BDNF) leading to interruption of long-term potentiation, a process responsible for spatial memory formation. This might be one of the most meaningful studies since it showed for the first time that the presence of sulfated heparan fragments in the plasma of septic patients correlated with cognitive impairment.

We also included three additional studies with a total of 140 patients that reported plasma values of glycoproteins ICAM-1, VCAM-1, P-selectin, and E-selectin. Specifically, Hamed et al. [56] and Su et al. [57] found increased levels of ICAM-1, VCAM-1, P-selectin, and E-selectin in SAE patients compared to sepsis patients. In contrast, one study, Tomasi et al. [58], showed no difference in ICAM-1 and VCAM-1 levels in SAE and sepsis patients.

Our analysis suggests that the circulating glycocalyx-associated products are elevated in sepsis patients with cognitive decline, and the extent of such marker production seems to be higher than that in sepsis patients without cognitive impairment. However, we acknowledge several limitations of the current meta-analysis. First, there is a limited number of studies addressing our research question. While our initial hypothesis involved proteoglycans, glycoproteins and glycosaminoglycans at equal importance, our search failed to identify sufficient studies specifically measuring proteoglycans; thus, we included studies that evaluated glycoproteins such as selectins and immunoglobulins. In addition, we suspect reporting bias, but we could not formally assess it due to the limited number of studies in this systematic review. Second, the differences in circulating levels among studies might be due to variations in disease severity, method of diagnosing SAE patients (GCS vs MoCA vs symptoms), and age of participants which may all have contributed to increased heterogeneity of the data. For example, Hamed et al. [56] evaluated pediatric patients with mean age of 4.31 years compared to adult patients in the other three studies [48, 57, 58]. Yet another reason for variations of the results may be due to timing of biomarker measurements. All studies report measuring biomarker levels during initial admission. However, the exact timing is not reported in any of the studies for comparison. Due to these limitations, it is difficult to conclude a threshold value for diagnosis of SAE. Specifically, one of the studies, Tomasi et al. [58], did not provide units for the reported ICAM-1 and VCAM-1 values, affecting the conclusions of our current analysis. Thirdly, the diagnostic specificity of these markers, especially in the case of adhesion glycoproteins such as ICAM-1, VCAM-1, E-selectin, and P-selectin, may be limited due to their shedding and production in other disease conditions such as diabetes mellitus, atherosclerosis, breast cancer, and Sjögren’s syndrome [59–63].

A significant strength of the current study is the identification of circulating glycocalyx products as a novel and potential serological biomarker for SAE independent of neuronal injury markers or cognitive assessments. Currently, neuronal biomarkers are being examined for the diagnosis of SAE. For example, Erikson et al. [64] found that delirium in septic shock patients was associated with an elevated S-100β, a cytoplasmic low molecular weight calcium-binding protein, when using a laboratory cutoff value of 0.15 μg/L. Another study determined that a neuron specific enolase concentration >12.5 μg/L was independently associated with a 23.3% (95% CI 6.7–39.9, P = .006) increased risk of 30-day mortality and a 29.3% (95% CI 8.8–49.8, P = .005) increased risk of delirium [65]. Other studies [54, 55] reported elevated glial fibrillary acidic protein and tau protein levels in SAE patients and proposed a serum cutoff value of 0.532 ng/ml and 75.92 pg/mL, respectively. However, most of these circulating neuronal degradation biomarkers are found to be elevated after cerebral injury has occurred, while glycocalyx degradation products may serve as early phase SAE biomarkers allowing time for clinical intervention.

## 5. Conclusion

To the best of our knowledge, this is the first systematic review to address the value of endothelial glycocalyx molecules for the diagnosis of SAE. Our study suggests that the glycosaminoglycan heparan sulfate and the glycoproteins ICAM-1, VCAM-1, and E-selectin are significantly increased in the plasma of sepsis patients with cognitive impairment. However, additional research is required to assess sensitivity, specificity, threshold values, and diagnostic utility of glycocalyx biomarkers. Future research on other glycocalyx components such as syndecans and chondroitin sulfate, as well as BBB-specific glycocalyx biomarkers, may further elucidate the role of glycocalyx degradation products in the diagnosis of SAE.

## Supporting information

S1 Table PRISMA checklist. (DOCX)

S1 File Search strategy. (DOCX)

S2 File Data extraction form for Hamed et al. (DOCX)

S3 File Data extraction form for Hippensteel et al. (DOCX)

S4 File Data extraction form for Su et al. (DOCX)

S5 File Data extraction form for Tomasi et al. (DOCX)
