# Supplementary material for: Endothelial glycocalyx-associated molecules as potential serological markers for sepsis-associated encephalopathy: A systematic review and meta-analysis
Source: PLoS One. 2023 Feb 21;18(2):e0281941. doi: 10.1371/journal.pone.0281941 (PMC9942976; doi:10.1371/journal.pone.0281941)
Supplement: S1 File — (DOCX) [file pone.0281941.s002.docx]

**PubMed search**

| **Search number** | **Search query** |
| --- | --- |
| 1 | "sepsis associated encephalopathy"[MeSH Terms] |
| 2 | "sepsis associated encephalopathy"[Title/Abstract] OR "SAE"[Title/Abstract] |
| 3 | "acute encephalopathy"[Title/Abstract] |
| 4 | "sepsis"[Title/Abstract] OR "septicemia*"[Title/Abstract] OR "bacteremia*"[Title/Abstract] OR "fungemia*"[Title/Abstract] OR "parasitemia*"[Title/Abstract] OR "viremia*"[Title/Abstract] OR "septic*"[Title/Abstract] |
| 5 | "encephalopathy"[Title/Abstract] OR "brain disorder"[Title/Abstract] OR "brain dysfunction"[Title/Abstract] OR "cognitive dysfunction"[Title/Abstract] |
| 6 | #4 AND #5 |
| 7 | "sepsis associated encephalopathy"[Title/Abstract] OR "sepsis induced brain dysfunction"[Title/Abstract] OR "acute brain dysfunction"[Title/Abstract] OR "sepsis associated delirium"[Title/Abstract] OR "sepsis induced cognitive impairment"[Title/Abstract] |
| 8 | #1 OR #2 OR #3 OR #6 OR #7 |
| 9 | "glycoproteins"[MeSH Terms] OR "glycoprotein*"[Title/Abstract] |
| 10 | "selectins"[MeSH Terms] OR "selectin*"[Title/Abstract] |
| 11 | "fibronectins"[MeSH Terms] OR "fibronectin*"[Title/Abstract] |
| 12 | "laminin"[MeSH Terms] OR "E-selectin"[Title/Abstract] OR "P-selectin"[Title/Abstract] OR "laminin*"[Title/Abstract] |
| 13 | "proteoglycans"[MeSH Terms] OR "proteoglycan*"[Title/Abstract] |
| 14 | "glycosaminoglycans"[MeSH Terms] OR "glycosaminoglycan*"[Title/Abstract] OR "GAG"[Title/Abstract] |
| 15 | "heparan sulfate proteoglycans"[MeSH Terms] OR "heparan sulfate"[Title/Abstract] |
| 16 | "chondroitin sulfates"[MeSH Terms] OR "chondroitin sulfate"[Title/Abstract] |
| 17 | "dermatan sulfate"[MeSH Terms] OR "dermatan sulfate"[Title/Abstract] |
| 18 | "hyaluronic acid"[MeSH Terms] OR "hyaluronate"[Title/Abstract] OR "hyaluronan"[Title/Abstract] OR "hyaluronic acid"[Title/Abstract] |
| 19 | "syndecans"[MeSH Terms] OR "syndecan*"[Title/Abstract] OR "endocan*"[Title/Abstract] |
| 20 | #9 OR #10 OR #11 OR #12 OR #13 OR #14 OR #15 OR #16 OR #17 OR #18 OR #19 |
| 21 | #8 AND #20 |

**EMBASE search**

| **Search number** | **Search query** |
| --- | --- |
| #1 | 'sepsis associated encephalopathy'/dm/exp OR 'sepsis associated encephalopathy' OR 'sepsis associated encephalopathy':ab,ti OR 'sae':ab,ti |
| #2 | 'brain disease'/dm OR 'brain disease':ab,ti OR 'encephalopathy':ab,ti OR 'brain dysfunction':ab,ti OR 'cognitive dysfunction':ab,ti OR 'cognitive defect':ab,ti OR 'cognitive disorder':ab,ti |
| #3 | 'sepsis'/dm OR 'sepsis':ab,ti OR 'septicemia':ab,ti OR 'bacteremia':ab,ti OR 'fungemia':ab,ti OR 'parasitemia':ab,ti OR 'viremia':ab,ti OR 'septic':ab,ti |
| #4 | #2 AND #3 |
| #5 | 'sepsis induced brain dysfunction':ab,ti OR 'acute brain dysfunction':ab,ti OR 'sepsis associated delirium':ab,ti OR 'sepsis induced cognitive impairment':ab,ti OR 'acute encephalopathy':ab,ti |
| #6 | #1 OR #4 OR #5 |
| #7 | 'glycoprotein'/exp OR glycoprotein:ab,ti |
| #8 | 'selectin glycoprotein'/exp OR selectin:ab,ti |
| #9 | 'padgem protein'/exp OR 'padgem protein':ab,ti OR 'p-selectin':ab,ti |
| #10 | 'endothelial leukocyte adhesion molecule 1'/exp OR 'endothelial leukocyte adhesion molecule 1':ab,ti OR 'e-selectin':ab,ti |
| #11 | 'l selectin'/exp OR 'l-selectin':ab,ti |
| #12 | 'fibronectin'/exp OR 'fibronectin':ab,ti |
| #13 | 'laminin'/exp OR 'laminin':ab,ti |
| #14 | 'proteoglycan'/exp OR 'proteoglycan':ab,ti |
| #15 | 'glycosaminoglycan'/exp OR 'glycosaminoglycan':ab,ti OR 'gag':ab,ti |
| #16 | 'heparan sulfate'/exp OR 'heparan sulfate':ab,ti |
| #17 | 'chondroitin sulfate'/exp OR 'chondroitin sulfate':ab,ti |
| #18 | 'dermatan sulfate'/exp OR 'dermatan sulfate':ab,ti |
| #19 | 'hyaluronic acid'/exp OR 'hyaluronic acid':ab,ti OR 'hyaluronate':ab,ti OR 'hyaluronan':ab,ti |
| #20 | 'syndecan'/exp OR 'syndecan':ab,ti |
| #21 | 'endocan'/exp OR 'endocan':ab,ti |
| #22 | #7 OR #8 OR #9 OR #10 OR #11 OR #12 OR #13 OR #14 OR #15 OR #16 OR #17 OR #18 OR #19 OR #20 OR #21 |
| #23 | #6 AND #22 |
