# Supplementary material for: Endothelial glycocalyx-associated molecules as potential serological markers for sepsis-associated encephalopathy: A systematic review and meta-analysis
Source: PLoS One. 2023 Feb 21;18(2):e0281941. doi: 10.1371/journal.pone.0281941 (PMC9942976; doi:10.1371/journal.pone.0281941)
Supplement: S2 File — (DOCX) [file pone.0281941.s003.docx]

**Data Extraction Form**

Author: Hamed, S. A.; Hamed, E. A.; Abdella, M. M.

Journal: Neuropediatrics Reviewer: Sheon Baby

Year: 2009

**STUDY CHARACTERISTICS**

**Study type:**

Randomized controlled trial (experimental study)

Cohort study (observational study)

Case-control study (observational study)

Other:

**Study arms:**

Single exposure arm *(i.e. 1 experimental and 1 control arm)*

Multi-arm Number of experimental arms: 3

**Study location:**

Single center

Name of institution: Assiut University Hospital, “the pediatric department, hospital of infectious diseases (fever hospital)” and “the pediatric clinic of the clinical pathology department”

Multicenter

Name of country:

**Study funding:**

Public (government)

Industry funded (industry provides all funding related to trial)

Industry sponsored (industry supplies materials used in the trial)

The study reports that no funding or support was received

Funding information was not reported

Other:

**Study objectives:**

**Primary: “**In this study, we hypothesized that measurement of the concentrations of some specific blood-derived and brain-derived proteins in the serum and cerebrospinal fluid (CSF) of patients with sepsis may have a diagnostic relevance in cases of encephalopathy induced by sepsis. We evaluated the concentrations of soluble intracellular adhesion molecule (sICAM-1) (marker of inflammation and endothelium-leukocyte interaction) [22, 33], nitric oxide (NO) and lipid peroxide (LPO) (markers for membrane lipid peroxidation) [3] and S-100B protein (brain-derived protein and a marker of astrocytes activation and injury) [37] in a group of children with sepsis with and without neurological manifestations and who were hemodynamically stable at presentation.”

**Number of patients enrolled:**

Control (no sepsis): 25

Non-exposed (sepsis alone): 24

Exposed (sepsis + encephalopathy): 16

**PATIENT CHARACTERISTICS**

**Age**

|  | Non-exposed arm | Exposed arm |
| --- | --- | --- |
| Mean | 51.75 months | |
| SD | 4.09 | |
| Range | 1-180 months | |

**Gender**

|  | Non-exposed arm | Exposed arm |
| --- | --- | --- |
| Male | 24 | |
| Female | 16 | |

**Setting**

|  | Control | Non-exposed arm | Exposed arm |
| --- | --- | --- | --- |
| Pediatric department | 0 | 40 | |
| Pediatric clinic of the clinical pathology department | 25 | 0 | |

**Etiology of sepsis**

|  | Non-exposed arm | Exposed arm |
| --- | --- | --- |
| *Streptococcus pneumoniae* | 24 | |
| *Staphylococcus aureus* | 10 | |
| *Escherichia coli* | 6 | |

**Disease severity**

|  | Non-exposed arm | Exposed arm |
| --- | --- | --- |
| Sepsis or severe sepsis | 40 | |
| Septic shock | 0 | |

**Patient characteristics at admission**

|  | Non-exposed arm | Exposed arm |
| --- | --- | --- |
| Admission time | 2.95 + 0.83 (mean)  1-5 days (range) | |
| Cough | n=20 (50%) | |
| Vomiting | n=18 (45%) | |
| Diarrhea | n=6 (15%) | |
| Bleeding tendency | n=6 (15%) | |
| Systolic BP | 99.20 + 18.60 mmHg (mean)  77-152 mmHg (range) | |
| Diastolic BP | 69.50 + 13.39 mmHg (mean)  45-92 mmHg (range) | |
| Temperature | 38.7 + 0.71^o^C (mean)  38-40^o^C (range) | |
| Pulse | 102.00 + 31.56 beats/min (mean)  61-183 beats/min (range) | |

**Neurological characteristics at admission**

|  | | Non-exposed arm | Exposed arm |
| --- | --- | --- | --- |
| Pediatric Glasgow Coma Scale | Mild | n=24 (60%) | |
|  | Moderate | n=12 (30%) | |
|  | Severe | n=4 (10%) | |
| Headache | | n=16 (40%) | |
| Drowsiness and lethargy | | n=12 (30%) | |
| Confusion | | n=12 (30%) | |
| Delirium and agitation | | n=8 (20%) | |
| Nuchal rigidity | | n=12 (30%) | |
| Seizures | | n=22 (55%) | |
| Paratonic rigidity | | n=6 (15%) | |
| Floppiness | | n=18 (45%) | |
| 6^th^ nerve palsy | | n=4 (10%) | |

**Neurological characteristics at discharge**

|  | Non-exposed arm | Exposed arm |
| --- | --- | --- |
| Mortality rate | n=2 (5%) | |
| Persistence of neurological manifestations | n=10 (25%)  Headache and drowsiness: n=8 (20%)  Rigidity of the 4 limbs: n=1 (2.5%)  Squint (6^th^ nerve palsy): n=4 (10%) | |
| No neurological abnormalities | n=28 (70%) | |

**Continuous data:**

Index: Serum ICAM-1 (ng/mL) (circle one: higher=better OR lower=better)

|  | Control | Non-exposed + Exposed arm | Non-exposed arm | Exposed arm |
| --- | --- | --- | --- | --- |
| Mean | 296.20 | 2240.19 | 2408.87 | 2895.22 |
| SD | 34.46 | 565.32 | 408.7 | 917.60 |
| Range | 227.00 – 340.00 | 1588.06 – 3069.59 | - | |
| p-value | P<0.0001 | | p<0.5 | |

Index: CSF ICAM-1 (ng/mL) (circle one: higher=better OR lower=better)

|  | Control | Non-exposed arm | Exposed arm |
| --- | --- | --- | --- |
| Mean | - | 45.15 | 65.65 |
| SD | - | 9.57 | 16.59 |
| p-value | - | p<0.5 | |

**Assessment of risk of bias (Case-control studies)**

**Selection**

1) Is the case definition adequate?

**a) Yes, with independent validation** (use of pediatric Glasgow coma scale)

b) Yes, for example, record linkage or based on self-reports

c) No description

2) Representativeness of the cases

**a) Consecutive or obviously representative series of cases**

b) Potential for selection biases or not stated

3) Selection of controls

a) Community controls

**b) Hospital controls**

c) No description

4) Definition of controls

**a) No history of disease (endpoint)**

b) No description of source

**Comparability**

1) Comparability of cases and controls on the basis of the design or analysis

**a) Study does not distinguish the characteristics of cases and controls.**

b) Study controls for any additional factor (These criteria could be modified to indicate specific control for a second important factor.)

**Exposure**

1) Ascertainment of exposure

**a) Secure record (e.g., surgical records)**

b) Structured interview where blind to case/control status

c) Interview not blinded to case/control status

d) Written self-report or medical record only

e) No description

2) Same method of ascertainment for cases and controls

**a) Yes**

b) No

3) Nonresponse rate

**a) Same rate for both groups**

b) Non-respondents described

c) Rate different and no designation
