# Supplementary material for: Endothelial glycocalyx-associated molecules as potential serological markers for sepsis-associated encephalopathy: A systematic review and meta-analysis
Source: PLoS One. 2023 Feb 21;18(2):e0281941. doi: 10.1371/journal.pone.0281941 (PMC9942976; doi:10.1371/journal.pone.0281941)
Supplement: S3 File — (DOCX) [file pone.0281941.s004.docx]

**Data Extraction Form**

Author: Hippensteel et al.

Journal: The Journal of Clinical Investigation Reviewer: Sheon Baby

Year: 2019

**STUDY CHARACTERISTICS**

**Study type:**

Randomized controlled trial (experimental study)

Cohort study (observational study)

Case-control study (observational study)

Other:

**Study arms:**

Single exposure arm *(i.e. 1 experimental and 1 control arm)*

Multi-arm Number of experimental arms:

**Study location:**

Single center

Name of institution: Hospital of the University of Pennsylvania

Multicenter

Name of country:

**Study funding:**

Public (government)

Industry funded (industry provides all funding related to trial)

Industry sponsored (industry supplies materials used in the trial)

The study reports that no funding or support was received

Funding information was not reported

Other:

**Study objectives:**

**Primary:** “We hypothesized that hippocampal penetration of circulating heparan sulfate fragments leads to sequestration of BDNF, impairing LTP and inducing septic cognitive impairment. In this report, we employed multimodal ex vivo, in vivo, and human studies to demonstrate that N- and 2-O- sulfated heparan sulfate fragments are released into the circulation during sepsis and inhibit BDNF-mediated hippocampal LTP, leading to cognitive dysfunction in both mice and humans.”

**Number of patients enrolled:**

Control: 9

Non-exposed (sepsis alone): 14

Exposed (sepsis + encephalopathy): 6

**PATIENT CHARACTERISTICS**

**Age**

|  | Non-exposed arm | Exposed arm |
| --- | --- | --- |
| Mean | 58 years | |
| Range | 41-64.5 years | |

**Gender**

|  | Non-exposed arm | Exposed arm |
| --- | --- | --- |
| Male | 12 | |
| Female | 8 | |

**Setting**

|  | Control | Non-exposed arm | Exposed arm |
| --- | --- | --- | --- |
| ICU | 0 | 20 | |
| Blood donors | 9 | 0 | |

**Etiology of sepsis**

|  | Non-exposed arm | Exposed arm |
| --- | --- | --- |
| Bacteria | - | |
| Virus | - | |
| Unclear | 20 | |

**Disease severity**

|  | Non-exposed arm | Exposed arm |
| --- | --- | --- |
| APACHE III score | 100 (93.5 – 124) | |
| Septic shock at presentation | 13 (65%) | |
| Vasopressor shock at presentation | 12 (60%) | |
| Any septic shock | 15 (75%) | |
| Acute respiratory failure at presentation | 12 (60%) | |
| Invasive ventilation at presentation | 6 (30%) | |
| Any invasive ventilation | 15 (75%) | |
| Acute respiratory distress syndrome | 10 (50%) | |
| Duration of mechanical ventilation (days) | 3 (1-7) | |
| ICU length of stay (days) | 6.5 (4.5-14) | |
| Montreal Cognitive Assessment Score (MoCA)* | 24 (22-25) | |
| Moderate/Severe Cognitive Impairment | 6 (30%) | |
| Trail Making Test Part B (seconds) | 95.9 (66.1 – 139.8) | |

*n=17, 3 patients unable to complete the MoCA due to severe cognitive impairment.

**Underlying disease**

|  | Non-exposed arm | Exposed arm |
| --- | --- | --- |
| Hypertension | 6 (30%) | |
| Diabetes | 6 (30%) | |
| Chronic kidney disease | 5 (25%) | |
| Congestive heart failure | 4 (20%) | |
| Cerebrovascular disease | 1 (5%) | |
| Cirrhosis | 1 (5%) | |
| Active malignancy | 9 (45%) | |
| Organ transplant | 7 (35%) | |
| Anxiety or depression | 5 (25%) | |

**Continuous data:**

Index: Plasma Heparan Sulfate Enriched in NS2S disaccharides (ng/mL) (circle one: higher=better OR lower=better)

|  | Non-exposed arm | Exposed arm | p-value |
| --- | --- | --- | --- |
| Median | 5.675 | 25.09 | 0.0478 |
| Range | 2.41-10.78 | 13.63-30.18 |  |

Index: Plasma Heparan Sulfate Enriched in NS + NS2S + NS6S disaccharides (ng/mL) (circle one: higher=better OR lower=better)

|  | Non-exposed arm | Exposed arm | p-value |
| --- | --- | --- | --- |
| Median | 23.665 | 90.665 | 0.0478 |
| Range | 11.21-31.85 | 39.65-136.87 |  |

**Assessment of risk of bias (Case-control studies)**

**Selection**

1) Is the case definition adequate?

**a) Yes, with independent validation**

b) Yes, for example, record linkage or based on self-reports

c) No description

2) Representativeness of the cases

**a) Consecutive or obviously representative series of cases**

b) Potential for selection biases or not stated

3) Selection of controls

a) Community controls

**b) Hospital controls**

c) No description

4) Definition of controls

**a) No history of disease (endpoint**)

b) No description of source

**Comparability**

1) Comparability of cases and controls on the basis of the design or analysis

**a) Study does not distinguish the characteristics of cases and controls.**

b) Study controls for any additional factor (These criteria could be modified to indicate specific control for a second important factor.)

**Exposure**

1) Ascertainment of exposure

**a) Secure record (e.g., surgical records)**

b) Structured interview where blind to case/control status

c) Interview not blinded to case/control status

d) Written self-report or medical record only

e) No description

2) Same method of ascertainment for cases and controls

**a) Yes**

b) No

3) Nonresponse rate

**a) Same rate for both groups**

b) Non-respondents described

c) Rate different and no designation
