# Supplementary material for: Endothelial glycocalyx-associated molecules as potential serological markers for sepsis-associated encephalopathy: A systematic review and meta-analysis
Source: PLoS One. 2023 Feb 21;18(2):e0281941. doi: 10.1371/journal.pone.0281941 (PMC9942976; doi:10.1371/journal.pone.0281941)
Supplement: S4 File — (DOCX) [file pone.0281941.s005.docx]

**Data Extraction Form**

Author: Su et al.

Journal: BioMed Research International Reviewer: Sheon Baby

Year: 2014

**STUDY CHARACTERISTICS**

**Study type:**

Randomized controlled trial (experimental study)

Cohort study (observational study)

Case-control study (observational study)

Other:

**Study arms:**

Single exposure arm *(i.e. 1 experimental and 1 control arm)*

Multi-arm Number of experimental arms:

**Study location:**

Single center

Name of institution: Kaoh siung Chang Gung Memorial Hospital (CGMH)

Multicenter

Name of country:

**Study funding:**

Public (government)

Industry funded (industry provides all funding related to trial)

Industry sponsored (industry supplies materials used in the trial)

The study reports that no funding or support was received

Funding information was not reported

Other:

**Study objectives:**

**Primary: “**This prospective study aimed to determine the roles of serum adhesion molecules and conventional biomarkers in predicting SE among adult severe sepsis patients.”

**Number of patients enrolled:**

Non-exposed (non-SE group): 47

Exposed (SE group): 23

**PATIENT CHARACTERISTICS**

**Age**

|  | Non-exposed | Exposed | p-value |
| --- | --- | --- | --- |
| Mean | 62.6 | 68.0 | 0.15 |
| SD | 13.6 | 11.2 |  |

**Gender**

|  | Non-exposed arm | Exposed arm | p-value |
| --- | --- | --- | --- |
| Male | 31 | 17 | 0.59 |
| Female | 16 | 6 |  |

**Setting**

|  | Non-exposed arm | Exposed arm |
| --- | --- | --- |
| ICU | - | - |
| Non-ICU | - | - |
| Emergency Room | 47 | 23 |

**Source of infection**

|  | Non-exposed arm | Exposed arm |
| --- | --- | --- |
| Respiratory tract infection | 19 | 8 |
| Urinary tract infection | 9 | 4 |
| Intra-abdominal infection | 10 | 4 |
| Soft tissue infection | 7 | 5 |
| Unknown origin | 2 | 1 |

**Concurrent bacteremia episode**

|  | Non-exposed arm | Exposed arm |
| --- | --- | --- |
| *Escherichia coli* | 6 | 5 |
| *Klebsiellapneumoniae* | 8 | 2 |
| *Proteus mirabilis* | 1 | 1 |
| *Burkholderiapseudomallei* | 0 | 1 |
| *Salmonella enteritidis* | 0 | 1 |
| *Streptococcus pneumoniae* | 2 | 0 |
| 𝛽*-Hemolytic Streptococcus group A* | 2 | 1 |
| *Staphylococcus aureus* | 2 | 2 |

**Disease severity**

|  | Non-exposed arm | | Exposed arm | | p-value |
| --- | --- | --- | --- | --- | --- |
|  | Mean | SD | Mean | SD |  |
| Maximum 24 h APACHE II score | 17.5 | 5.7 | 21.3 | 5.5 | 0.01 |
| CCI score | 3.2 | 3.0 | 3.6 | 2.6 | 0.44 |
| Maximum 24 h SOFA  score | 5.4 | 3.1 | 8.2 | 2.4 | <0.001 |

**Patient characteristics at admission**

|  | Non-exposed arm | | Exposed arm | | p-value |
| --- | --- | --- | --- | --- | --- |
|  | Mean | SD | Mean | SD |  |
| Systolic BP | 108.0 | 47.0 | 108.0 | 39 | 0.91 |
| Diastolic BP | 64.2 | 22.8 | 72.1 | 24.2 | 0.20 |
| Pulse rate | 112 | 25 | 103 | 26 | 0.26 |
| Respiratory rate | 21 | 4 | 20 | 3 | 0.25 |
| Shock within 24 hrs | 34 (74%) | - | 15 (65%) | - | 0.58 |
| Ventilator treatment within 24 hrs | 11 (24%) | - | 13 (57%) | - | 0.008 |

**Underlying disease**

|  | Non-exposed arm | Exposed arm | p-value |
| --- | --- | --- | --- |
| Diabetes mellitus | 15 (32%) | 10 (44%) | 0.43 |
| Hypertension | 22 (47%) | 8 (35%) | 0.44 |
| Liver disease/alcoholism | 6 (13%) | 6 (26%) | 0.19 |
| Chronic lung disease | 11 (23%) | 3 (13%) | 0.36 |
| Stroke | 4 (9%) | 7 (30%) | 0.03 |
| Coronary artery disease | 6 (13%) | 0 (0%) | 0.17 |
| Cancer | 10 (21%) | 6 (21%) | 0.76 |
| Chronic renal disease | 1 (2%) | 2 (9%) | 0.25 |

**Continuous data:**

Index: ICAM-1 (ng/mL) (circle one: higher=better OR lower=better)

| Non-exposed arm | | Exposed arm | | p-value |
| --- | --- | --- | --- | --- |
| Mean | SD | Mean | SD |  |
| 764.8 | 504.9 | 1028.2 | 525.2 | 0.03 |

Index: VCAM-1 (ng/mL) (circle one: higher=better OR lower=better)

| Non-exposed arm | | Exposed arm | | p-value |
| --- | --- | --- | --- | --- |
| Mean | SD | Mean | SD |  |
| 1969.0 | 1129.5 | 3048.1 | 1261.1 | 0.001 |

Index: P-selectin (ng/mL) (circle one: higher=better OR lower=better)

| Non-exposed arm | | Exposed arm | | p-value |
| --- | --- | --- | --- | --- |
| Mean | SD | Mean | SD |  |
| 98.0 | 23.0 | 98.8 | 42.5 | 0.77 |

Index: E-selectin (ng/mL) (circle one: higher=better OR lower=better)

| Non-exposed arm | | Exposed arm | | p-value |
| --- | --- | --- | --- | --- |
| Mean | SD | Mean | SD |  |
| 117.8 | 127.6 | 214.2 | 111.9 | 0.22 |

**Assessment of risk of bias (Case-control studies)**

**Selection**

1) Is the case definition adequate?

**a) Yes, with independent validation** (Symptoms of SE included somnolence, stupor, coma, confusion, disorientation, agitation, irritability, and decreased level of GCS. Encephalopathy was confirmed if the patient had two or more of the aforementioned symptoms for more than 72 hours, regained consciousness after treatment, or deteriorated and died.)

b) Yes, for example, record linkage or based on self-reports

c) No description

2) Representativeness of the cases

**a) Consecutive or obviously representative series of cases**

b) Potential for selection biases or not stated

3) Selection of controls

a) Community controls

**b) Hospital controls**

c) No description

4) Definition of controls

**a) No history of disease (endpoint)**

b) No description of source

**Comparability**

1) Comparability of cases and controls on the basis of the design or analysis

**a) Study controls for underlying diseases** (Select the most important factor.)

b) Study controls for any additional factor (These criteria could be modified to indicate specific control for a second important factor.)

**Exposure**

1) Ascertainment of exposure

**a) Secure record (e.g., surgical records)**

b) Structured interview where blind to case/control status

c) Interview not blinded to case/control status

d) Written self-report or medical record only

e) No description

2) Same method of ascertainment for cases and controls

**a) Yes**

b) No

3) Nonresponse rate

**a) Same rate for both groups**

b) Non-respondents described

c) Rate different and no designation
