# Supplementary material for: Endothelial glycocalyx-associated molecules as potential serological markers for sepsis-associated encephalopathy: A systematic review and meta-analysis
Source: PLoS One. 2023 Feb 21;18(2):e0281941. doi: 10.1371/journal.pone.0281941 (PMC9942976; doi:10.1371/journal.pone.0281941)
Supplement: S5 File — (DOCX) [file pone.0281941.s006.docx]

**Data Extraction Form**

Author: Tomasi et al.

Journal: Molecular neurobiology Reviewer: Sheon Baby

Year: 2017

**STUDY CHARACTERISTICS**

**Study type:**

Randomized controlled trial (experimental study)

Cohort study (observational study)

Case-control study (observational study)

Other:

**Study arms:**

Single exposure arm *(i.e. 1 experimental and 1 control arm)*

Multi-arm Number of experimental arms: 3

**Study location:**

Single center

Name of institution: São José Hospital

Multicenter

Name of country:

**Study funding:**

Public (government)

Industry funded (industry provides all funding related to trial)

Industry sponsored (industry supplies materials used in the trial)

The study reports that no funding or support was received

Funding information was not reported

Other: Department of Psychiatry and Behavioral Sciences, The University of Texas Health Science Center at Houston (UTHealth) Medical School.

**Study objectives:**

**Primary:** “In this pilot trial, we hypothesized that in non-severe septic patients, blood biomarkers of inflammation, endothelial activation, coagulation, and brain function would be different when compared to patients with and without brain dysfunction.”

**Number of patients enrolled:**

Control (no sepsis): 8

Non-exposed (sepsis alone): 20

Exposed (sepsis + encephalopathy): 10

**PATIENT CHARACTERISTICS**

**Age**

|  | All patients | Control | Non-exposed | Exposed | p-value |
| --- | --- | --- | --- | --- | --- |
| Median | 60 | 57 | 63 | 53 | 0.68 |
| Range | 29-88 | 38-88 | 39-74 | 29-78 |  |

**Gender**

|  | All patients | Control | Non-exposed | Exposed | p-value |
| --- | --- | --- | --- | --- | --- |
| Male | 19 | 6 | 7 | 6 | 0.12 |
| Female | 19 | 2 | 13 | 4 |  |

**Setting**

|  | All patients | Control | Non-exposed | Exposed | p-value |
| --- | --- | --- | --- | --- | --- |
| ICU admission | 4 | 0 | 0 | 4 | 0.002 |
| Non-ICU admission | 34 | 8 | 20 | 6 |  |

**Etiology of sepsis**

|  | Control | Non-exposed arm | Exposed arm |
| --- | --- | --- | --- |
| Bacteria | N/A | - | - |
| Virus | N/A | - | - |
| Unclear | N/A | 20 | 10 |

**Disease severity – CURB 65 Score**

|  | All | Control | Non-exposed arm | Exposed arm | p-value |
| --- | --- | --- | --- | --- | --- |
| Median | N/A | N/A | 2 | 2 | 0.47 |
| Range | N/A | N/A | 2-4 | 2-3 |  |

**Continuous data:**

Index: ICAM-1 (circle one: higher=better OR lower=better)

|  | All | Control | Non-exposed | Exposed | p-value |
| --- | --- | --- | --- | --- | --- |
| Median | 85,722 | 52,081 | 108,280 | 90,044 | 0.029 |
| Range | 611–180,641 | 611–89,200 | 12,275–180,641 | 63,222–126,868 |  |

Index: VCAM-1 (circle one: higher=better OR lower=better)

|  | All | Control | Non-exposed | Exposed | p-value |
| --- | --- | --- | --- | --- | --- |
| Median | 589,516 | 516,867 | 674,231 | 592,802 | 0.022 |
| Range | 1308–1,189,201 | 1308–678,275 | 349,032–1,189,201 | 559,826–974,631 |  |

**Assessment of risk of bias (Case-control studies)**

**Selection**

1) Is the case definition adequate?

**a) Yes, with independent validation**

b) Yes, for example, record linkage or based on self-reports

c) No description

2) Representativeness of the cases

**a) Consecutive or obviously representative series of** cases

b) Potential for selection biases or not stated

3) Selection of controls

a) Community controls

**b) Hospital controls**

c) No description

4) Definition of controls

**a) No history of disease (endpoint)**

b) No description of source

**Comparability**

1) Comparability of cases and controls on the basis of the design or analysis

**a) Study controls for disease severity (Select the most important factor.)**

b) Study controls for any additional factor (These criteria could be modified to indicate specific control for a second important factor.)

**Exposure**

1) Ascertainment of exposure

**a) Secure record (e.g., surgical records)**

b) Structured interview where blind to case/control status

c) Interview not blinded to case/control status

d) Written self-report or medical record only

e) No description

2) Same method of ascertainment for cases and controls

**a) Yes**

b) No

3) Nonresponse rate

**a) Same rate for both groups**

b) Non-respondents described

c) Rate different and no designation
